# A low-cost method to rapidly and accurately screen for transpiration efficiency in wheat

**DOI:** 10.1186/s13007-018-0339-y

**Published:** 2018-08-29

**Authors:** Andrew Fletcher, Jack Christopher, Mal Hunter, Greg Rebetzke, Karine Chenu

**Affiliations:** 1 Queensland Alliance for Agriculture and Food Innovation (QAAFI), The University of Queensland, 203 Tor Street, Toowoomba, QLD 4350 Australia; 2 Queensland Alliance for Agriculture and Food Innovation (QAAFI), The University of Queensland, 13 Holberton Street, Toowoomba, QLD 4350 Australia; 3 School of Agriculture and Food Sciences, The University of Queensland, St Lucia, 4072 Australia; 4 CSIRO Plant Industry, PO Box 1600, Canberra, ACT 2601 Australia

**Keywords:** Water use efficiency, Water use, Transpiration, Phenotyping platform, Crop adaptation, Crop improvement, Breeding, Drought, Water deficit, Water stress, Wheat, *Triticum aestivum*, Cereal

## Abstract

**Background:**

Wheat (*Triticum aestivum* L.) productivity is commonly limited by the availability of water. Increasing transpiration efficiency (biomass produced per unit of water used, TE) can potentially lead to increased grain yield in water-limited environments (‘more crop per drop’). Currently, the ability to screen large populations for TE is limited by slow, low-throughput and/or expensive screening procedures. Here, we propose a low-cost, low-technology, rapid, and scalable method to screen for TE. The method uses a Pot-in-Bucket system that allows continuous watering of the pots and frequent monitoring of water use. To investigate the robustness of the method across environments, and to determine the shortest trial duration required to get accurate and repeatable TE estimates in wheat, plants from 11 genotypes varying in phenology were sown at three dates and grown for different durations in a polyhouse with partial environmental control.

**Results:**

The method revealed significant genotypic variations in TE among the 11 studied wheat genotypes. Genotype rankings for TE were consistent when plants were harvested the same day, at the flag-leaf stage or later. For these harvests, genotype rankings were consistent across experiments despite changes in environmental conditions, such as evaporative demand.

**Conclusions:**

These results indicate that (1) the Pot-In-Bucket system is suitable to screen TE for breeding purposes in populations with varying phenology, (2) multiple short trials can be carried out within a season to allow increased throughput of genotypes for TE screening, and (3) root biomass measurement is not required to screen for TE, as whole-plant TE and shoot-only TE are highly correlated, at least in wheat. The method is particularly relevant in developing countries where low-cost and relatively high labour input may be most applicable.

**Electronic supplementary material:**

The online version of this article (10.1186/s13007-018-0339-y) contains supplementary material, which is available to authorized users.

## Background

Water availability is one of the primary limiting factors of yield for bread wheat (*Triticum aestivum* L.). With projected increase in water-stress events in some regions due to climate change [1, 2] and continuing global population growth, greater food production is needed. This can be achieved, in part, through greater crop yield and more efficient use of limited resources, such as water. Transpiration efficiency (TE), which is defined as the amount of biomass produced per unit of water transpired, has been suggested as a trait of interest to improve yield in drought-prone environments, in particular where crops rely on stored soil moisture [3–5]. In such environments, crops that are able to utilise available soil water more efficiently can maintain greater soil water reserves early during the crop cycle to use later in the season, when water can be more effectively used to produce grain yield (e.g. [6, 7]). In Australia, increases in yield with the release of new wheat cultivars has been linked with increases in TE [8], suggesting that indirect selection for TE has occurred as a by-product of breeding for yield in the drought-prone environments of the Australian wheatbelt [9]. Crop simulation studies have also indicated major potential yield gains related to an increase in TE or its component traits [5, 10, 11]. Further, experimental work on carbon-13 isotope discrimination (CID) in leaf dry biomass, a surrogate trait for TE, has highlighted the potential of TE to increase yield in wheat [12]. Selection based on CID has resulted in the release of two high water-use efficiency cultivars in Australia, Drysdale and Rees [4, 13].

TE of a plant is typically expressed as grams of biomass produced (with or without the roots) per kilogram of water transpired. It is commonly measured in sealed pots that exclude soil evaporation and deep drainage. TE differs from ‘water use efficiency’ (WUE), a term used in agronomy and ecology, which typically refers to field measurements of biomass per unit of initial soil water plus in crop rainfall and any irrigation applied but which does not account for soil water evaporation, deep drainage and excludes root biomass [14]. TE can also be defined at the leaf level, and then corresponds to the ratio between carbon assimilation (photosynthesis) and water flux through the stomata. Leaf TE is commonly measured using gas exchange with point measurements in terms of both space (part of a leaf) and time (seconds to minutes). As a result, TE measured on individual leaves is typically more variable than plant-level measurements [15]. Even with multiple leaf-level point measurements per plant in stable light conditions, TE measured on parts of single leaves may still be poorly correlated with whole-plant TE [15]. Overall, measurements on individual leaves are generally less suitable for large-scale phenotyping than plant-level measurements.

While genomics has the potential to accelerate crop adaptation, one of the current greatest bottlenecks is arguably the ability to phenotype large numbers of genotypes [16] for traits of importance for target production environments such as TE [3, 17]. TE at the plant level can be phenotyped with gravimetric methods in pots, by estimating the amount of water transpired by changes in pot weight over time. In this case, the watering and weighing can be performed manually [18] or with automated platforms [19–21]. TE can also be phenotyped indirectly, in either pot or field experiments, using CID measurements on dried laminas typically harvested between the ‘stem elongation’ stage and flowering [12]. In C3 species such wheat, CID is a stable trait that negatively correlates with TE [12, 22, 23]. In comparison to direct measurements of whole-plant TE that requires dedicated experiments, CID can be measured from samples harvested in field trials, such as breeding trials. However it is also more expensive [24] and its relationship with TE in C4 plants is not as straight forward as in C3 plants [25, 26].

Here, we propose a method for screening a large number of plants for TE quickly, accurately and efficiently with a low-cost platform that can be easily scalable. While measurements of whole-plant TE are typically done around flowering [8, 27], this paper demonstrates that reliable screening of TE can be achieved with shorter trial duration, to allow multiple trials to be carried out in a season and thus increase the throughput of whole-plant TE phenotyping.

## Methods

### Overview

**Fig. 1:**
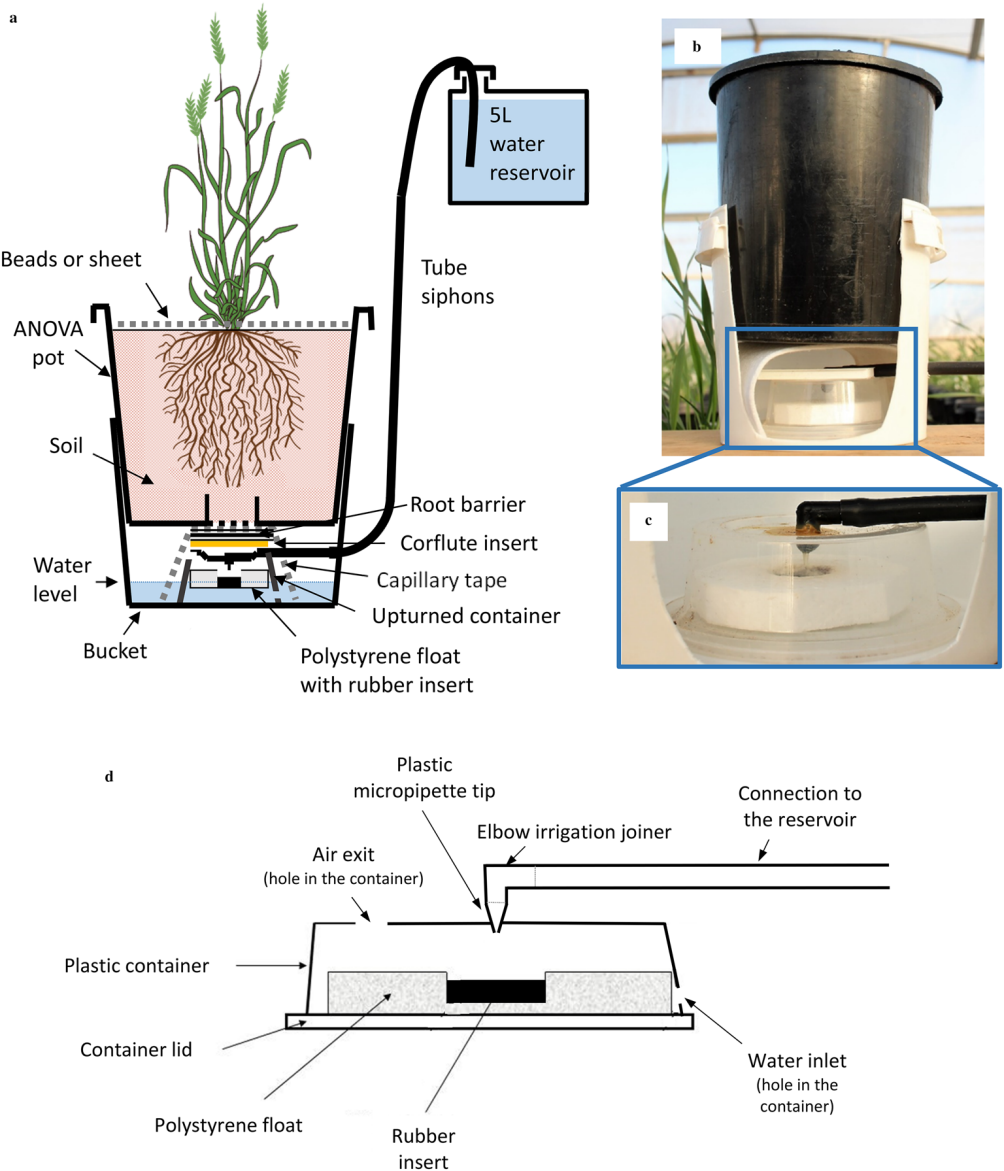
‘Pot in Bucket’ system. **a** A schematic diagram of the system, **b** a photograph where the bucket has a section removed to exhibit the capillarity mat that allows water to be taken up from the bucket to the soil in the pot, as well as the float valve, which controls the water level in the bucket, and **c** a photograph and **d** diagram of the float valve, where the micropipette tip can be blocked by the polystyrene float to stop the water flow from the water reservoir, and thus controls the water level in the bucket. Details of the system and how to construct it are presented in Additional file 1: Figure S1 and Additional file 2

Three experiments were conducted with 11 wheat genotypes grown in a pot system that maintains a constant water table and allows recording of water loss over time (Fig. 1 and Additional file 1: Figure S1). Above-ground and root biomass were measured to determine TE for the shoot and the whole plant, respectively. In the first experiment, six harvests were performed at regular intervals from 40 days after sowing (i.e. at stage ‘6 visible leaves’ on average across genotypes) to 14 days after flowering (on average across all genotypes) in order to identify the shortest period required to get consistent genotypic discrimination for TE. To ensure the repeatability of the method, another two consecutive experiments were conducted with later sowings, where TE measurements occurred at the shortest time identified in the first experiment.

### The Pot-in-Bucket system

The ‘Pot in Bucket’ system (Fig. 1 and Additional file 1: Figure S1; Additional file 2) was adapted from the system described by Hunter et al. [28, 29]. It consists of (1) a pot of soil where the plant or plants are grown, which is connected with (2) a capillary mat to (3) a bucket where the water level is kept constant due to (4) a float valve, itself connected to (5) a water reservoir via (6) a tube (Fig. 1; details for the construction can been seen in Additional file 1: Figure S1 and in a demonstration video, Additional file 2). For wheat, the pots used were 1.4 L ANOVApot^®^ (137 mm top diameter, 116 mm base diameter, 140 mm height, http://www.anovapot.com/php/anovapot.php), specifically designed to reduce the escape of roots through the pot drainage hole. However, other types of pots could be used in the system, as a root barrier is placed under the pot to stop roots from growing outside of the pot (Fig. 1a and Additional file 1: Figure S1F). The base of the pot, with its grid-covered central hole, rests on a piece of capillary mat draped over a small container that sits inside a bucket (Fig. 1b), A float valve contained within the small container (Fig. 1c) maintains a constant water table within the bucket providing water to the upper pot via capillary flow to the central hole and the soil medium. The valve is connected via a standard medical IV drip with a 200 µm filter to a 5L container (reservoir), which is sealed to avoid any evaporation yet loose enough to prevent vacuum forming (Fig. 1a). Hence, water use from plant transpiration and soil evaporation corresponds to the amount of water removed from the reservoir. The measurement of water loss can thus be done by either weighing the reservoir to see how much water has been removed, or by weighing the amount of water required to refill the reservoir to a pre-defined level, which can be faster.

### Materials and growing conditions

All three experiments were conducted in a shade house, with solarweave covers that exclude rain in Gatton, Queensland, Australia (− 27°55′41.4″S, 152°33′94.4″E). Eleven wheat genotypes were grown in the experiments: (1) seven varieties from varying genetic backgrounds (Babax, Drysdale, Hartog, Mace, Scout, Seri M82 and Suntop), which were known to contrast for TE and vary for phenology; and (2) four near isogenic lines (NILs) contrasting in CID to test the ability of the system to phenotype changes in TE which are likely to be more subtle. The NILs were produced following an initial cross of the low CID variety Quarrion to high CID variety Hartog and then three further rounds of crossing to the recurrent parent Hartog with selection of backcross-derived progeny for CID. In total, four rounds of crossing to Hartog produce BC3-derived NILs varying for CID. In previous well-managed, irrigated field experiments, NIL11 and NIL28 exhibited low CID and are expected to have a high TE; while NIL63, and NIL113 exhibited high CID, and are expected to have a low TE.

The 11 genotypes were sown on May 6 (Exp. 1), June 16 (Exp. 2) and July 14 (Exp. 3) 2015 using five pots per genotype and two plants of the same genotype per pot (four seeds sown and thinned to two plants). Plants were grown using the PIB system with unrestricted access to water in a red silty-clay soil collected from the Redland Bay Department of Agriculture and Fisheries Research Station (27°31′42.4″S 153°14′47.2″E). The soil was mixed thoroughly with 2.8 g/L of Osmocote “All Purpose” fertiliser (NPK of 21.2:1.9:5.7). When seedlings had emerged, a 2 cm layer of white polyurethane beads was applied to the soil surface in each pot (between 7 to 14 days after sowing) to reduce moisture loss through evaporation, as well as inhibiting weed growth.

### Design

A complete randomized block design was used having blocks of 55 pots, each block consisting of five-pot replicates of each genotype, was used for each harvesting time and each experiment (Table 1). Pots used for measurements were surrounded by border pots to reduce ‘border effects’ [30]. For each experiment, an additional three pots with no plants were used to measure any background moisture loss (e.g. remaining soil evaporation).

**Table 1:** Experiment characteristics, including sowing and harvest dates, trial duration (in days and in degree days (°Cd) after sowing), environmental conditions and plant characteristics at harvest

Exp.	Harvest	Sowing date	Harvest date	Trial duration (days)	Trial duration (°Cd)	Mean temp. (°C)	Mean VPD (kPa)	Leaf area (cm^2^ plt^−1^)	Mean Zadoks stage	Above-ground biomass at harvest (g)	Cumulative transpiration (mL)
1	E1H1	06/05/2015	15/06/2015	40	677	21.7	1.70	52 ^(46)^ ^[36–68]^	15^(21) [15–16]^	0.85^(0.85)^ ^[0.73–0.92]^	97^(95)^ ^[70–117]^
	E1H2		29/06/2015	54	912	21.4	1.58	166^(149)^ ^[101–212]^	17^(22)^ ^[16–18]^	1.12^(0.96)^ ^[0.71–1.37]^	282^(257)^ ^[211–337]^
	E1H3		13/07/2015	68	1127	21.2	1.54	306^(318)^ ^[209–380]^	39^(43)^ ^[37–43]^	3.03^(3.85)^ ^[1.86–3.89]^	615^(705)^ ^[400–780]^
	E1H4		28/07/2015	83	1352	20.6	1.46	430^(427)^ ^[326–503]^	58^(60)^ ^[50–62]^	7.3^(7.22)^ ^[4.90–9.44]^	1240^(1205)^ ^[829–1532]^
	E1H5		12/08/2015	98	1572	20.9	1.54	316^(291)^ ^[166–454]^	>70^(>70)^ ^[>70]^	10.2^(10)^ ^[5.28–13.88]^	1801^(1761)^ ^[916–2346]^
	E1H6		18/08/2015	104	1664	21.1	1.59	286^(219)^ ^[207–364]^	> 70^(>^ ^70)^ ^[> 70]^	13.5^(11)^ ^[10.88–16.14]^	2486^(2005)^ ^[1960–3117]^
2	E2H1	16/06/2015	25/08/2015	70	1079	21.3	1.68	335^(249)^ ^[249–471]^	46^(45)^ ^[39–52]^	4.00^(2.86)^ ^[2.86–5.98]^	992^(751)^ ^[699–1321]^
3	E3H1	14/07/2015	10/09/2015	58	905	23.9	2.18	254^(335)^ ^[185–335]^	42^(45)^ ^[39–44]^	3.00^(3.8)^ ^[1.66–3.80]^	797^(1056)^ ^[529–1056]^

Mean daily temperature and mean daylight vapour pressure deficit (VPD) were calculated over the period used to estimate TE (i.e. from the beginning to the end of water use measurements for each harvest). Harvests are labelled by experiment (E1–E3) and the harvest within each sowing (H1–H6). Mean Zadoks stage, leaf area, and above-ground dry biomass at harvest, as well as the cumulative amount of water transpired are given (1) for the trial mean (i.e. mean of all 11 genotypes), (2) for the reference cv. Hartog (in brackets) and (3) for the range of genotypic mean values (in square brackets). Note that in E1H1, no significant difference was found among genotypes, therefore, results from this harvest are presented in Table 2, but not elsewhere in the paper nor included in most analyses

### Environmental measurements

Air temperature (T_air_) and atmospheric relative humidity (RH) were measured using a Vaisala HMP60 sensor (Vaisala, Helsinki, Finland), and light radiation was measured using a radiation sensor (Apogee Instruments, Providence, Utah USA) at 2 m from the soil, recording every minute and averaged every 10 min. vapour pressure deficit (VPD) was calculated as follows [31]:

1 $${\text{VPD}} = 0.61078\,*\,\left( {1 - {\text{RH}}/100} \right)*\exp \left( {17.269\,*\,{\text{Tair}}/\left( {{\text{Tair}} + 237.3} \right)} \right).$$

### Plant measurements

Measurements of plant water use were made weekly from 25 days after sowing (stage ‘4 visible leaves’) until harvest. Prior to 25 days, soil imbibition with water (after sowing), soil evaporation (mainly before the addition of a layer of plastic beads on soil surface) and very small transpiration rate from seedlings prevented accurate and reliable measurements of transpiration.

Weekly observations of plant development were made on the main stem of one plant from each of the pots of the last-harvested block from experiment 1 (E1H6) and of all of the pots from experiments 2 and 3 (E2H1 and E3H1). Measurements included the number of visible green leaves, number of dead leaves, tiller number, stem number, and Zadoks score for the main stem [32].

Plants were harvested approximately every fortnight from 40 days after sowing in Experiment 1, with the exception of the final harvest, which occurred 6 days after the previous harvest (i.e. 14 days after the trial-average of the main-stem flowering time; Table 1). In Experiments 2 and 3, all plants were harvested approximately 1000 °Cd (degree-days with a base temperature of 0 °C) after sowing, i.e. after the flag leaf was fully expanded for most genotypes (Table 1).

At each harvest, plants were cut at the soil level and dissected into the main plant components: green leaf blades, senesced blades, stems with leaf sheaths, and spikes. Leaf area of the green blades was measured using a leaf area meter (LI-3100C; LI-COR, Inc., Lincoln, Nebraska USA). Roots were collected by carefully removing the soil substrate using a hose fitted with a fine spray head and gently teasing the roots apart. Due to the highly dispersible nature of the soil used in this experiment, the roots did not require excessive manipulation to remove soil particles, which can often result in inadvertent loss of roots. Once separated, all plant material was oven dried at 70 °C for five days, and then weighed to record dry biomass.

### Estimation of transpiration efficiency

Transpiration efficiency was calculated as the ratio of dry (unless otherwise stated, e.g. in Additional file 1: Figure S4) biomass per cumulative gram of water transpired from 25 days after sowing until harvest. The cumulative transpiration per pot was calculated as the total amount of water removed from each water reservoir, minus the average cumulative water lost from the three water loss control pots without plants, in each experiment for the period considered.

### Data analysis

T-tests were applied to determine confidence interval and compare genotypic differences (*P* = 0.05). Least significant differences (LSD) were calculated to group genotypes with similar TE (*P* = 0.05). A series of bivariate linear models were performed to estimate phenotypic correlations between experiments and harvests, thus providing a measure of the strength of the agreement in genotype rankings between experiments and harvests. All data analyses were performed using the R software environment [33].

## Results

### Similar variability for shoot and whole-plant transpiration efficiency

**Fig. 2:**
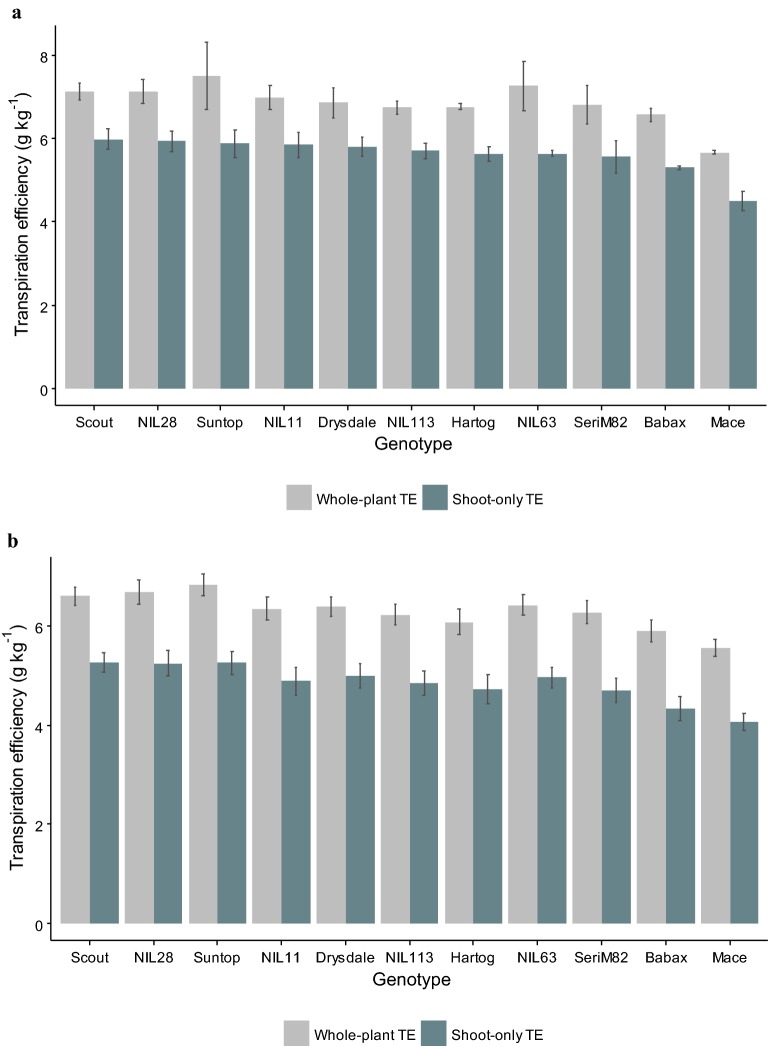
Whole-plant (shoots plus roots) and shoot-only transpiration efficiency for all 11 genotypes studied. Data for **a** harvest 8 days after the trial-mean flowering date of the main head in experiment 1 (E1H5), and for **b** all harvests and experiments (excluding E1H1). Error bars represent confidence interval at *P *= 0.05 (n = 5)

In cereals, transpiration efficiency (TE) is typically measured at flowering or soon after, prior to the main period of leaf senescence. When harvested 8 days after the trial-average flowering time of the main head (E1H5), the 11 studied genotypes had a relatively wide range of TE, with shoot TE (i.e. excluding the roots) varying from 4.5 to 6 g kg^−1^, and whole-plant TE ranging from 5.6 to 7.5 g kg^−1^ (Fig. 2). Significant differences (*P *<0.05) among genotypes were found for both whole-plant and shoot-only TE, with Scout, Suntop, NIL 28 and NIL 11 having the greatest TE, while Babax, and particularly Mace, were the less water efficient (Fig. 2).

**Fig. 3:**
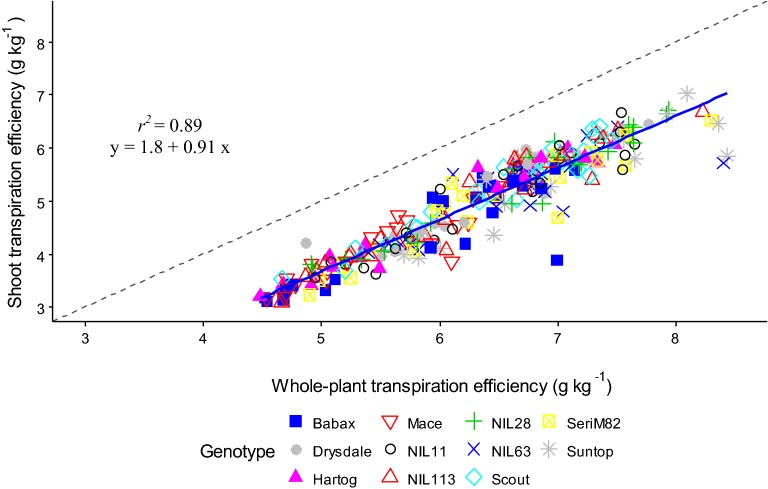
Whole-plant transpiration efficiency versus shoot transpiration efficiency for all 11 genotypes studied. The figure includes individual-pot data from all the harvests and experiments, except the two earliest harvests from Experiment 1 (E1H1 and E1H2), for which biomass accumulation and water use were low, and the root biomass harder to measure accurately. For information, when only data from E1H1 was excluded the relationship had a *r*^2^ of 0.815. The 1:1 relationship is represented by the hatched line

Whole-plant and shoot TE were highly correlated (*r* = 0.94; Fig. 3) and the ranking of genotypes for the two traits was generally consistent across harvests and experiments. As whole-plant and shoot TE were so closely correlated, results for shoot TE only are presented elsewhere.

### Genotype ranking for transpiration efficiency is relatively stable when harvesting occurs at or following the flag-leaf stage

**Fig. 4:**
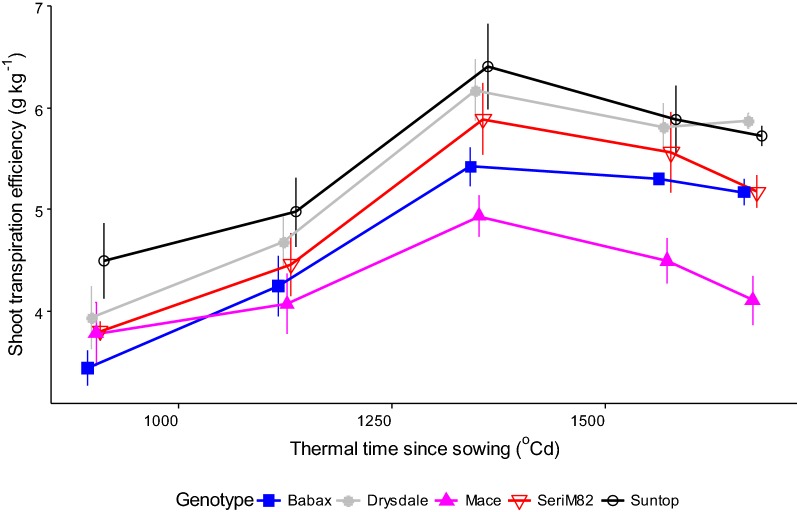
Genotypic variations in shoot transpiration efficiency over thermal time. Data for all harvests of Experiment 1, with the exception of harvest 1 (E1H1), for which the small size of the plants (6-leaf stage for the trial average) and their limited water use make differences in TE difficult to capture. For clarity, only five of the 11 genotypes were presented in this figure. Error bars represent confidence interval at *P* = 0.05 (n = 5). See Additional file 1: Figure S2 for all genotypes

In the first experiment (Fig. 4, Table 2), TE across genotypes was analysed for harvests from the trial-mean 6-leaf stage (E1H1) to 14 days after trial-mean flowering time for the main head (E1H6), i.e. before major leaf senescence. At the 6-leaf stage (E1H1), genotypic differences for TE were mostly not significant (Table 2), most likely due to the small plant size and their limited water use, which made differences in TE difficult to capture. As a result, data from this first harvest (E1H1) were excluded from the analysis. The first significant genotypic differences in TE were detected 912 °Cd after sowing (E1H2), at the trial-mean 8-leaf stage (Zadoks stages varied from 16 to 18 among genotypes).

**Table 2:** Mean shoot transpiration efficiency (g kg^−1^) for all 11 studied genotypes for the six harvests of Experiment 1

Genotype	Mean shoot TE (g kg^−1^)					
	E1H1	E1H2	E1H3	E1H4	E1H5	E1H6
Suntop	12.05^a^	4.49^a,b^	4.98^b,c^	6.40^a^	5.89^a,b^	5.72^b,c^
NIL28	9.78^b^	4.07^c,d^	5.57^a^	6.37^a^	5.94^a,b^	6.10^a^
Scout	9.02^b,c^	4.75^a^	5.27^a,b^	5.95^b,c^	5.99^a^	5.58^c,d^
Drysdale	9.55^b^	3.94^c,d,e^	4.68^c,d^	6.17^a,b,c^	5.81^a,b,c^	5.87^a,b^
NIL11	9.64^b^	3.35^g^	5.22^a,b^	6.07^a,b,c^	5.86^a,b,c^	5.65^b,c,d^
Hartog	9.42^b^	3.60^e,f,g^	5.60^a^	5.99^b,c^	5.64^b,c,d^	5.49^c,d^
NIL63	8.52^b,c^	4.15^b,c^	4.95^b,c^	5.96^b,c^	5.64^b,c,d^	5.44^d^
SeriM82	9.66^b^	3.80^d,e^	4.46^d,e^	5.89^c^	5.56^c,d^	5.17^e^
NIL113	7.84^b,c^	3.88^c,d,e^	4.73^c,d^	6.23^a,b^	5.71^a,b,c^	5.65^b,c,d^
Mace	8.72^b,c^	3.78^d,e,f^	4.07^e^	4.93^e^	4.50^e^	4.11^f^
Babax	7.40^c^	3.44^f,g^	4.25^e^	5.42^d^	5.30^d^	5.17^e^

Superscript letters represent genotype groups that were not significantly different based on least significant difference (LSD, *P* = 0.05)

Genotype rankings were relatively stable for TE for all the harvests from flag leaf (i.e. Zadoks 37–39) to 14 days after the trial-mean flowering time of the main head (Table 2, Fig. 4 and Additional file 1: Figure S2; E1H3-E1H6). TE measured at harvest 2 (E1H2) had a correlation coefficient of only 0.38 with the harvest performed soon after flowering (E1H5; Table 3). By contrast, correlation coefficients of harvest 3 (E1H3) onwards with the reference harvest E1H5 near flowering were above 0.7 (Table 3). This suggests that while significant differences in TE can be detected around the 8-leaf stage, 912 °Cd after sowing (E1H2), better discrimination among genotypes can be achieved 2 weeks later, 1127 Cd after sowing (E1H3), from the flag-leaf stage onwards.

**Table 3:** Correlations between mean shoot transpiration efficiency measured for different harvests from each experiment

	E1H1	E1H2	E1H3	E1H4	E1H5	E1H6	E2H1	E3H1
E1H1	1.00	0.39	0.38	0.52	0.39	0.33	0.56	0.52
E1H2	0.39	1	0.25	0.35	0.38	0.27	0.79	0.57
E1H3	0.38	0.25	1	0.69	0.73	0.72	0.60	0.56
E1H4	0.52	0.35	0.69	1	0.92	0.94	0.73	0.79
E1H5	0.39	0.38	0.73	0.92	1	0.95	0.73	0.79
E1H6	0.33	0.27	0.72	0.94	0.95	1	0.65	0.79
E2H1	0.56	0.79	0.60	0.73	0.73	0.65	1	0.85
E3H1	0.52	0.57	0.56	0.79	0.79	0.79	0.85	1

Overall, genotype rankings for TE were similar, regardless of time of measurement from harvest 2 (E1H2) to 6 (E1H6). However, a time greater than approximately 1000 °Cd (i.e. flag-leaf stage) was required for TE to be highly correlated with TE measured around flowering.

### Ranking of genotypes is maintained across environments for early estimates of transpiration efficiency

**Fig. 5:**
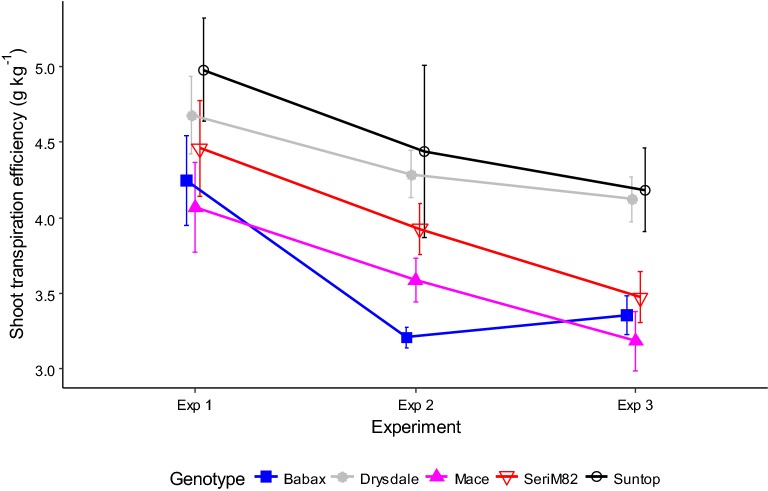
Genotypic variations in shoot transpiration efficiency measured at ~ 1000 °Cd after sowing, across experiments. Measurements for three experiments (i.e. E1H3, E2H1 and E3H1) are shown. For clarity, only five of the 11 genotypes were presented in this figure. Error bars represent confidence interval at *P *=* 0.*05 (n = 5). See Additional file 1: Figure S3 for all genotypes

Two experiments were performed with later sowings to assess how stable genotype rankings are across environments, when harvest occurs after the flag-leaf stage, around 1000 °Cd after sowing. Lower TE (Fig. 5) was observed in both later-sown experiments (E2H1 and E3H1) compared to TE measured at a similar phenological stage in Experiment 1 (E1H3), likely due to greater evaporative demand (i.e. higher VPD) in these later-sown experiments (Table 1). As in Experiment 1, significant genotypic differences were observed in experiments 2 and 3 (E2H1 and E3H1; Table 4 and Fig. 5). Importantly, genotypic values of TE in Experiments 2 and 3 were significantly correlated with those from Experiment 1, and in particular with the reference harvest, which occurred soon after the trial-mean flowering time of the main head (E1H5) (correlation of 0.73 and 0.79, respectively; Table 3). Note that E3H1 (harvested at 905 °Cd after sowing) was strongly correlated with TE measured around flowering (*r* = 0.79; E1H5) while E1H2, which was harvested in similar conditions (harvested at 912 °Cd) was not (*r* = 0.38), thus illustrating the impact that environments can have on the results. In this case, plants from E1H2 had experienced a lower VPD than E3H1 plants on average (Table 1) and particularly towards time of harvest when growth rate and transpiration rate were at their maximum (data not shown).

**Table 4:** Mean shoot transpiration efficiency (g kg^−1^) for all 11 studied genotypes for harvests soon after the flag-leaf stage, around 1000 °Cd after sowing, in the three experiments (i.e. E1H3, E2H1 and E3H1)

Genotype	Mean shoot TE (g kg^−1^)		
	Experiment 1 (E1H3)	Experiment 2 (E2H1)	Experiment 3 (E3H1)
Suntop	4.98^b,c^	4.44^a,b^	4.18^a^
NIL28	5.57^a^	4.42^a,b^	3.97^a,b^
Scout	5.27^a,b^	4.61^a^	3.97^a,b^
Drysdale	4.68^c,d^	4.29^b,c^	4.12^a,b^
NIL11	5.22^a,b^	4.02^c,d^	3.92^b^
Hartog	5.60^a^	3.81^d,e^	3.54^c,d^
NIL63	4.95^b,c^	4.24^b,c^	4.13^a,b^
SeriM82	4.46^d,e^	3.93^d^	3.47^c,d^
NIL113	4.73^c,d^	4.02^c,d^	3.68^c^
Mace	4.07^e^	3.59^e^	3.19^e^
Babax	4.25^e^	3.21^f^	3.36^d,e^

Superscript letters represent genotype groups that were not significantly different based on least significant difference (LSD, *P* = 0.05)

### Transpiration efficiency can be estimated over shorter periods if estimated later in the crop cycle

Transpiration efficiency was also estimated between the different harvests to look at the accuracy of such estimates (Table 5). High correlations of TE with the reference (i.e. TE measured soon after the main-head flowering, E1H5) were consistently found for water use measurements taken over 3 weeks or more. This indicates that it is possible to only measure water use and plant growth over a short period of time to identify line differences in TE, as long as (1) the plants are big enough (here from harvest 1 onwards, i.e. from the 6-leaf stage) and (2) plant biomass is estimated at the beginning of the measurement period.

**Table 5:** Correlations between (1) the shoot transpiration efficiency calculated between harvests from the first experiment (first column), and (2) the reference shoot transpiration efficiency, i.e. for a harvest performed soon after flowering (E1H5)

Considered period	Duration of the considered period (days)	TE correlation with E1H5
E1H1–E1H2	14	− 0.13
E1H2–E1H3	14	0.73
E1H3–E1H4	15	0.13
E1H4–E1H5	15	0.16
E1H5–E1H6	6	0.18
E1H1–E1H3	28	0.63
E1H2–E1H4	29	0.77
E1H3–E1H5	30	0.90
E1H4–E1H6	21	0.83
E1H3–E1H6	36	0.91
E1H1–E1H4	43	0.90
E1H2–E1H5	44	0.94
E1H1–E1H5	58	0.97
E1H2–E1H6	50	0.92
E1H1–E1H6	64	0.92

The duration between the considered harvests is presented in days. For each considered period, TE of each genotype was calculated as the difference of the genotypic mean biomass between the final harvest and initial harvest, divided by the genotypic mean water use between those two harvests (n = 11)

### Can the Pot-in-Bucket method be used for non-destructive measurement of biomass and transpiration efficiency?

Non-destructive estimates of root and shoot fresh biomass and TE were tested against measurements from harvested plants to assess their accuracy. In the Pot-In-Bucket system, pot weights were recorded (1) initially, after full wetting of the soil; (2) at harvest with the plant in the pot; and (3) at harvest after cutting the above-ground biomass to estimate the fresh biomass of the roots (3–1), shoots (2–3) and whole plants (2–1), as well as TE for fresh biomass. Such whole-pot estimates for root, above-ground, and whole-plant biomass were correlated both with (1) fresh biomass (e.g. for E1H6: roots, *r* = 0.71; shoots, *r* = 0.95; whole-plant, *r* = 0.94; Additional file 1: Figures S4A, C and E), and (2) dry biomass (e.g. for E1H6: roots, *r* = 0.65; shoots, *r* = 0.95; whole-plant, *r* = 0.91; Additional file 1: Figures S4B, D and F) at each particular stage (data only shown for E1H6).

By contrast, estimates for TE derived from pot weight were poorly correlated with the ones estimated from measured plant biomass (*r* < 0.24 for whole plant TE; Additional file 1: Figure S4G and H). Hence, in the conditions tested, the accuracy of TE estimates derived from pot weight was insufficient to compare genotypes.

## Discussion

### Is it necessary to include root biomass when ranking genotypes for transpiration efficiency?

In the conditions tested in this study, TE for whole-plant biomass (including roots) was closely correlated with TE for above-ground biomass from the 8-leaf stage onwards (Fig. 3; *r* = 0.94). Similar results have been found in other conditions in wheat and in sorghum [*Sorghum bicolor* (L.) Moench] [3]. Root extraction is a labour-intensive process, and it often results in small amounts of fine roots being lost during washing, introducing error into root biomass estimates [34] and thus also whole-plant TE estimates. While the Pot-in-Bucket system presented here allows estimation of whole-plant biomass from pot weight measurement over time (Additional file 1: Figure S4; [18]), those estimates were not precise enough to provide useful estimates of TE in the conditions tested (Additional file 1: Figure S4G-H). Overall, given the high correlation between shoot and whole-plant TE and the practical difficulties that come with root-biomass estimation, shoot TE appears as an appropriate target for genetic and breeding purposes.

However, genotypic differences in biomass partitioning to roots have been observed under well-watered conditions in crops such as wheat, sorghum and maize (*Zea mays* L.), which means that the exclusion of the roots could potentially affect the ranking of genotypes for TE [3]. In the present study, some variation in genotype ranking between shoot TE and whole-plant TE was observed for specific conditions (Fig. 3; data not presented). In sorghum, significant genotype by environment (G × E) interactions have been reported for shoot TE, but not for whole-plant TE, thus illustrating the potential importance of including roots in the calculation of TE [35]. Hence, considering root biomass may be important for detailed physiological studies.

### Do environmental conditions and trial duration affect genotype ranking for transpiration efficiency?

Trial duration and growing conditions affected TE (Figs. 4, 5). While TE estimates changed over time (Fig. 4 and Additional file 1: Figure S2), genotype rankings for TE remained consistent from the flag-leaf stage (E1H3) through to 2 weeks after flowering (last studied harvest) in Experiment 1 (E1H6; Table 3 and Fig. 4 and Additional file 1: Figure S2). This suggests that a trial should run at least up to the flag-leaf stage (i.e. 900–1000 °Cd after sowing; but before major leaf senescence) to allow accurate screening of TE in wheat.

The studied genotypes varied for phenology (Table 1). In the proposed method, all genotypes were harvested at a common date for each harvest (reported stages in the text are given for the average across genotypes). By doing so, genotypes grew in the same environmental conditions, and did not experience different environmental conditions towards harvest, when plants are the biggest and transpire the most, i.e. when environmental conditions have the greatest impact on TE. That said, TE of genotypes harvested at the same stage (flowering) or at the same date (at flag leaf) were also relatively highly correlated (0.57) in a set of genotypes varying by 30 days for flowering (Chenu and Fletcher, unpublished data). Overall, harvesting all genotypes at the same time is simpler than targeting specific stages, and it allowed genotype ranking to be maintained across experiments. Thus, the proposed method was found suitable for screening genotypes with different phenology.

In terms of the effect of environmental conditions, TE tended to be lower for higher VPD conditions (e.g. for similar stages: E2H1 and E3H1 compared to E1H3; Table 1 and Fig. 5). Importantly however, the genotype rankings observed in all of these conditions were highly correlated (Table 3). Other studies have reported non-significant G × E interactions for TE under well-watered conditions ([36] for rice (*Oryza sativa* L.); [37], [38] and [3] for sorghum). Even in studies where significant G × E interactions for TE were observed, these interactions were still smaller than the genotypic main effect (in peanut (*Arachis hypogea* L.) [39]; in sorghum [35] and [40]). Thus, the method proposed should be suitable for other species as well.

Genotypic TE differences were enhanced in Experiments 2 and 3 (Fig. 5 and Additional file 1: Figure S3), probably because of greater VPD. This agrees with previous reports of more detailed analyses of VPD effects, which have shown that increased VPD tends to increase TE differences among genotypes [3, 20]. Given the response of transpiration rate to VPD [41], low VPD conditions and/or small plant size (e.g. E1H2, E1H3) result in limited transpiration, while higher VPD conditions typically result in increased transpiration (e.g. with a lower leaf area, plants in E3H1 (higher VPD) transpired more than larger plants in E1H3 (lower VPD) at a similar developmental stage; Table 1). In addition, genetic variation for transpiration rates is typically greater under high than under low VPD (e.g. [20, 41]), possibly resulting in higher genetic variations for TE [3, 20]. Hence, high VPD conditions may allow earlier discrimination of the genotypic variability for TE.

Measuring biomass accumulation and water use for shorter windows of time later in the vegetative period, did not substantially affect the genotype ranking for TE. Hence, TE measurements could be made over shorter time periods (21 days minimum here) if plant biomass is estimated at the beginning of the period (Table 5).

Overall, while sowing time did affect the absolute value of TE, it had little impact on genotypic rankings. Importantly, it was identified that a minimum period of about 900–1000 °Cd (flag-leaf stage) is required to identify genotypic differences in TE. Furthermore, discrimination for TE among genotypes was enhanced under higher VPD conditions.

### An interesting method to assist breeding for drought-prone regions

After the flag-leaf stage, the genotype ranking for TE is relatively stable. While trials to phenotype TE typically run up to flowering or a bit longer (e.g. [3, 8]), shortening the trial duration up to the flag-leaf stage means that, at least in wheat, multiple experiments may be conducted in the same space within a single season, greatly increasing the potential throughput of a screening platform. In this study, trial duration was shortened by a third compared to the reference period to near flowering (E1H3 vs E1H5), so that two experiments (Experiments 1 and 3) could easily be carried out within the usual Australian wheat growing season.

The Pot-in-Bucket system allowed accurate non-destructive estimations of plant biomass (Additional file 1: Figure S4A-F) by measuring the increase in pot weight (i.e. difference between an empty pot with fully-wetted soil, and the same pot with the grown plant). However, those non-destructive biomass estimates did not result in accurate estimations of TE (Additional file 1: Figure S4G-H). Doing the measurements early morning before shoot water content drops could improve the results [42, 43]. Alternatively, shoot biomass could be estimated via image analysis (e.g. [19, 21]). A method allowing non-destructive measurements of biomass (and thus TE) would allow an integrative phenotyping-breeding process, with TE being phenotyped at the flag leaf, and plant crossed later, at flowering (e.g. [44]).

While the Pot-in-Bucket system does not allow screening under limited soil–water conditions, the system has value for plant breeding for drought-prone regions as TE in well-watered and drought-stressed conditions have been found positively correlated (*P* < 0.01) in wheat [45]. In these circumstances, screening under favourable conditions could be preferable as it maximises variation among genotypes and increases heritability [46]. In addition, the Pot-in-Bucket system can be set up in controlled environments to study tolerance to high temperature and/or elevated CO_2_. Hence, the Pot-in-Bucket system appears appropriate (1) for breeding for drought-prone regions, particularly where crops rely heavily on stored soil water [3, 5, 11, 13], and (2) to study impacts of factors associated with global warming on TE.

### A screening method for low-resource breeding programs

The Pot-in-Bucket system is a low-cost, low-technology method that can be scaled up to do high-throughput phenotyping of TE in wheat or other crops with particular relevance to developing countries, such as rice and barley or bigger crops such as maize and sorghum, when using bigger ANOVApot^®^ (van Oosterom, unpublished data). While expensive high-throughput systems requiring state-of-the-art technology are appropriate for measuring TE in developed countries or in international research institutes (e.g. Australia [3]; Europe [20]; ICRISAT [19]), these technologies are not appropriate for crop improvement programs where resources and technologies are more limited. Cheaper phenotyping platforms such as the one proposed by Pereyra-Irujo et al. [21] can be manufactured, but they still require a certain level of engineering.

The Pot-In-Bucket system presented here is a transferable concept that does not require any specific skilled labour and could readily be implemented anywhere in the world (Additional file 1: Figure S1). The technology is simple and cost effective, enabling the system to be built and maintained effectively. The system is however relatively labour intensive for its construction, maintenance and usage. For example, in this study, monitoring and recording of water use required one person for 1 day per week, and the harvesting of plants was also manual. The method thus appears be particularly effective for small experiments in most countries, and for medium-to-high throughput experiments where the cost of labour is relatively low. Cheap, low-technology and scalable methods, such as clear-pot root phenotyping platform [47] have already successfully been transferred to developing countries. By leveraging the low cost of labour, the Pot-In-Bucket system could be effectively deployed in developing nations to enable crop improvement programs to screen the extent of genetic diversity for TE in their germplasm collections, ultimately enabling the mapping of genes associated with TE.

## Conclusions

Harvests of wheat genotypes differing in phenology were performed every fortnight in a Pot-in-Bucket system to identify the minimum period required to get accurate and robust estimates of transpiration efficiency (TE). Measuring TE at early stages of plant development (before 8-leaf stage) did not allow confident discrimination among genotypes, as the small amount of water used and small plant biomass produced led to poor TE estimates. Only when harvesting at or after the flag-leaf stage, was significant variation in TE detected with relatively consistent genotype ranking. Environmental conditions resulting from different sowing times affected the absolute value of TE but had little influence on the genotype ranking for TE. Genotypic discrimination for TE was enhanced in high VPD conditions. Furthermore, measuring root biomass did not appear to be necessary to estimate TE for genetic and breeding purposes, as whole-plant TE and shoot TE were highly correlated.

The shortening of TE-screening trials can allow multiple trials to be run in a single season, which can effectively multiply the throughput of any given TE phenotyping platform.

The low-cost, low-technology and high-throughput Pot-in-Bucket method should enable breeding programs with limited resources, e.g. in developing countries, to screen their germplasm for variation in TE to improve drought adaptation in wheat and other crops.

## Additional files

**Additional file 1.** Supplementary information.

**Additional file 2.** Video on how to construct the Pot-In-Bucket system.
