# Supplementary material for: A low-cost method to rapidly and accurately screen for transpiration efficiency in wheat
Source: Plant Methods. 2018 Aug 29;14:77. doi: 10.1186/s13007-018-0339-y (PMC6116455; doi:10.1186/s13007-018-0339-y)
Supplement: Supplementary file 1 — Additional file 1. Supplementary information. [file 13007_2018_339_MOESM1_ESM.docx]

**Supplementary Figures**

**Figure S1 Steps to construct the low-cost transpiration efficiency system.** Individual and semi-assembled components (A-G) used for the Pot-In-Bucket system (H).

Some of the materials used in this instructional figure can be substituted based on availability as long as the basic principles of the system are met (i.e. a constant water table is maintained, water is able to move freely into the pot via capillary action, and water use can be measured by the amount of water removed from a sealed container).

A video can be viewed to watch the main steps of the Pot-In-Bucket construction in Additional file 2.

Materials required:

- Plastic pot, e.g. ANOVA® pot that helps securing the roots within the pot ([www.anovapot.com](http://www.anovapot.com))
- Plastic bucket (e.g. white bucket in G) with roughly the same diameter as the plastic pot to minimize water loss from evaporation between the two containers
- Plastic container (e.g. small plastic takeaway container displayed in A)
- 1 cm thick polystyrene sheet (used in B)
- 5 mm thick rubber mat (used in B)
- 5 mm thick corflute sheet (D)
- Capillary matting (3-5 mm thick; E)
- Micropipette tips
- 5 mm garden irrigation elbow piece (with barbs)
- 5 mm garden irrigation hose
- 5 mm medical IV line (preferably with translucent plastic to enable checking for the presence of unwanted bubbles)
- Rigid sheet of thin plastic (less than 1 mm thick)
- Sealed water container (e.g. 5L jar; H)
- Water-proof glue (e.g. a contact adhesive and a bathroom sealant)

Constructing the Pot-in-Bucket system (Fig 2), including the float valve (A and C):

1. Trim the barb off one end of the 5 mm garden irrigation elbow piece
2. Cut the micropipette tips at 5 mm from the tip at a 45^o^ angle. Insert this into the trimmed elbow piece and glue in place using a small amount of contact adhesive (ensuring that there is no room for water to leak past the tip), with the face of the 45^o^ cut directed toward the untrimmed barb to ensure the flow of water is not blocked (C).
3. Cut a 10-15 cm length of the 5 mm garden irrigation hose. Insert the barbed end of the elbow piece into one end of the hose.
4. Drill three 5 mm holes in the small plastic container (circled in red in A): 1 centre of the base (to put the elbow assembly in), 1 offset on the base (to let the air pass), 1 in the side as close to the lip of the container as possible (to let the water pass).
5. Insert the elbow assembly into the centre hole with the micropipette tip facing down and glue in place using contact adhesive.
6. Cut a disc (or hexagon in B) in the polystyrene (ensure that it can move freely inside the plastic container and can float up to the glued micropipette tip when water will be added, but not so small that it will drift inside the container once filled with water). Using a 10 mm hole punch, cut a hole through the centre of the polystyrene disc (B).
7. Using the same 10 mm hole punch, cut a disc in the rubber mat. Insert a rubber disc horizontally into the 10 mm hole in the polystyrene and glue in place using the bathroom sealant (N.B. If the polystyrene disc is too small, the rubber disc may be misaligned with the micropipette tip and not stop the flow of water as required; A).
8. Cut a square of corflute to roughly the size of the plastic container. Remove a thin section from the centre to allow the corflute to shroud the extruding elbow piece and hose (D). This corflute square will sit above the small plastic container, and protect the elbow and hose from the weight of the pot with the soil and the plant (G, Figure 1B).
9. Cut a square of rigid plastic material to the same size as the corflute. This will sit about the corflute and below the capillary mat to avoid root extension (E). This part is actually optional.
10. Cut a strip of the capillary material (F) long enough to sit over the valve, corflute and plastic square (in an upside down ‘U’ shape), with both ends reaching the base of the float container to ensure maximum contact with the water (G).
11. Drill a hole in the side of the plastic bucket which is to be used as the reservoir. The diameter of the hole needs to the same as the garden irrigation hose. Insert the valve into the bucket, feeding the hose through the hole until the float sits in the centre (G).
12. Remove the plastic mesh from the ANOVA® covering the drainage hole at the base of the pot (remove by pressing the mesh out with your thumbs, or carefully using a knife or scissors). Cut a small square of capillary matting to cover this hole and glue it outside of the pot to ensure best contact between the two pieces of capillarity mat and the soil (N.B. use enough glue to ensure that this piece of capillary mat will not fall off, but be careful not to cover with glue the capillary matting covering the drainage hole itself , as this could reduce water flow). The pot will then sit on top of the valve assembly, inside the bucket (H, Fig 2B)
13. Cut the gardening irrigation hose so that when inserted into the water jug it can reach the bottom, and allow around 5-10 cm extra for a better manoeuvrability of the system. Fit this onto the translucent end of the medical IV line and ensure it will not leak.
14. Drill a hole in the cap of the water bottle at the same diameter as the irrigation hose. Feed the hose through the hole to the depth of the bottle (it should be very tight to prevent evaporation), allowing the extra 5-10 cm to dangle with the translucent plastic.
15. At the beginning of the experiment, fill the water bottle and make sure the water level inside the jar is above the level of the valve (to allow a syphon). Insert the IV/hose line and screw down cap (H - N.B. do not over tighten the cap, some air will need to get in to allow water to syphon out).
16. Suck on the other end of the IV line to start a water syphon. Once water is flowing freely, connect the IV line to the hose protruding from the reservoir and valve (H). Adjust the water level in the jar to a pre-established level (or fill to top) when ready to record water use.

Figure S2 Shoot transpiration efficiency over thermal time for all 11 genotypes studied. Data for all harvests of experiment 1, with the exception of harvest 1 (E1H1), for which the small size of the plants (6-leaf stage) and their limited water use made differences in TE difficult to capture. Error bars represent confidence interval at *P* = 0.05 (n = 5).

Figure S3 Genotypic variations in shoot transpiration efficiency measured at ~1000^o^Cd after sowing for all 11 studied genotypes. Measurements for all three experiments (E1H3, E2H1, E3H1). Error bars represent confidence interval at *P* = 0.05 (n = 5).

**Figure S4 Comparisons of plant biomass and transpiration efficiency from (non-destructive) pot weight versus direct biomass measurements.** Pot weight measurements allow plant biomass to be derived non-destructively, where direct plant biomass measurement require destructive sampling. A) Fresh root biomass derived from pot weights versus measured fresh biomass of washed roots. B) Derived fresh root biomass versus measured dry root biomass. C) Derived fresh shoot biomass versus measured fresh shoot biomass. D) Derived fresh shoot biomass versus measured dry shoot biomass. E) Derived fresh plant biomass versus measured fresh plant biomass. F) Derived fresh plant biomass versus measured dry plant biomass. F) Transpiration efficiency for measured fresh plant biomass versus transpiration efficiency for derived fresh plant biomass. G) Transpiration efficiency for measured dry whole-plant biomass versus transpiration efficiency for derived fresh whole-plant biomass. Data is presented for all 11 genotypes for E1H6. Derived fresh whole-plant biomass transpiration efficiency was calculated as accumulated biomass over time (the difference in pot weights between initial and final measurements) per unit of water transpired for the same period. Fresh whole-plant transpiration efficiency was calculated as total fresh plant material (roots and shoots) weighed at harvest per unit of water transpired for the same period. The 1:1 relationship is represented by the hatched line.

**Figure S1 Steps to construct the low-cost transpiration efficiency system.**


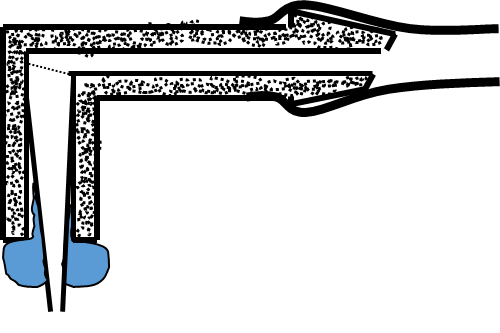

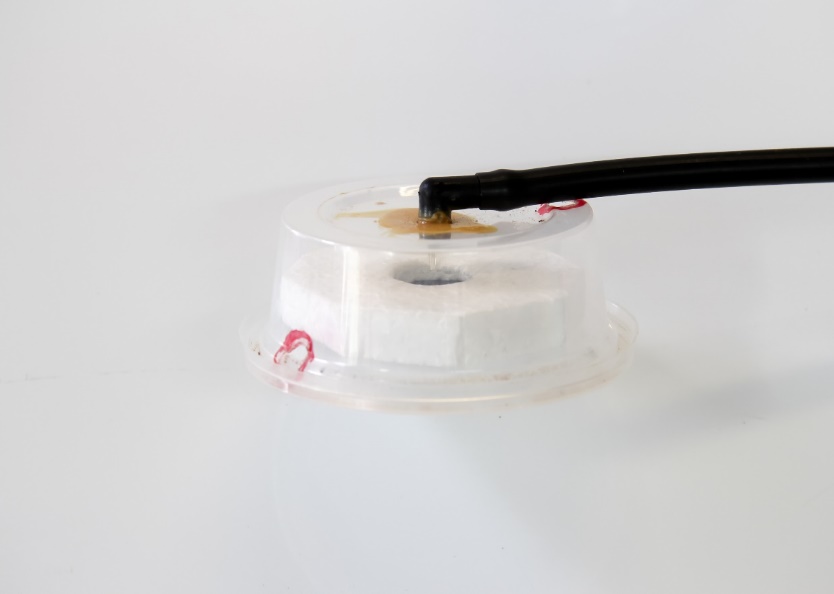

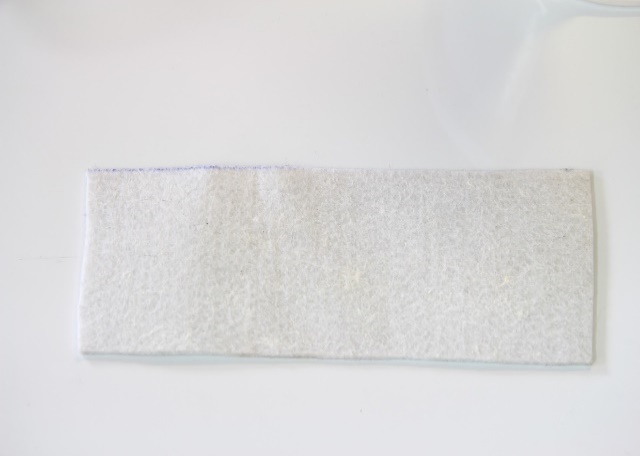

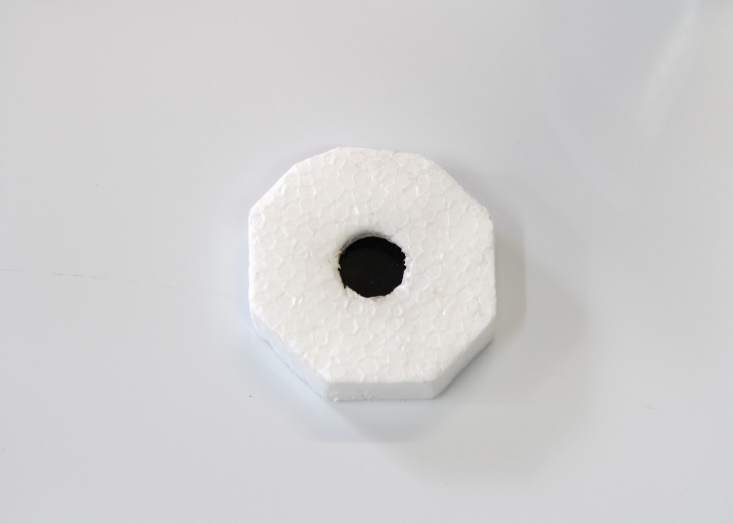

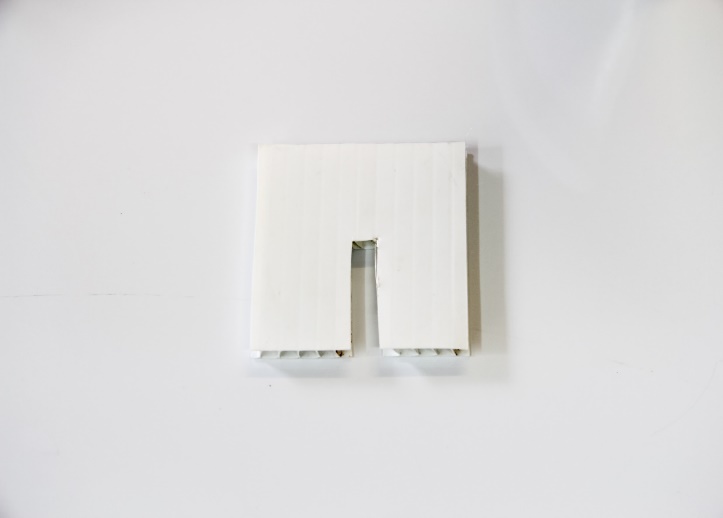

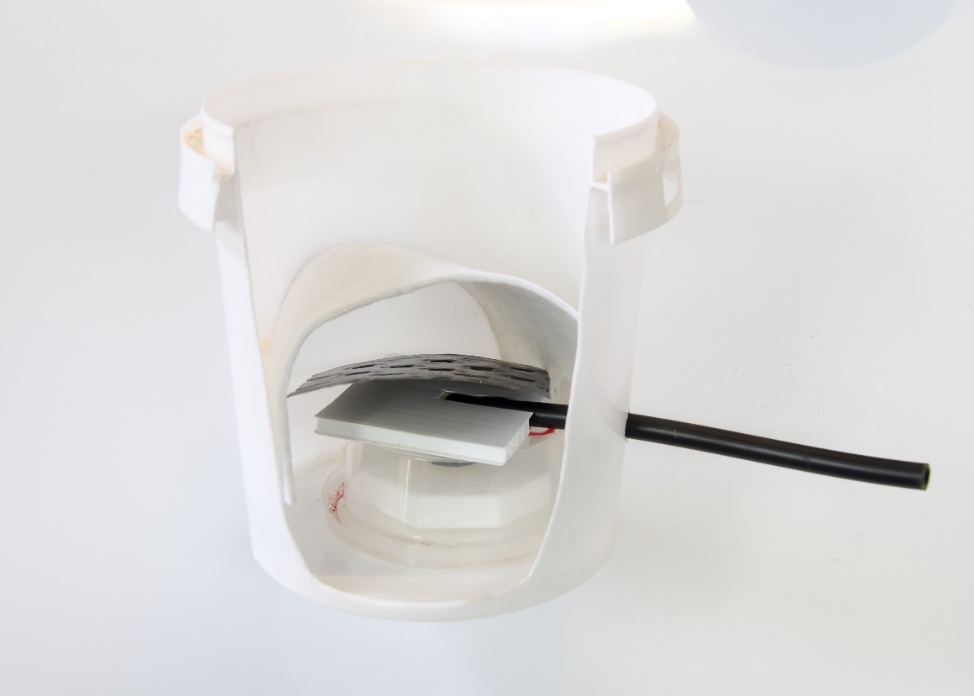

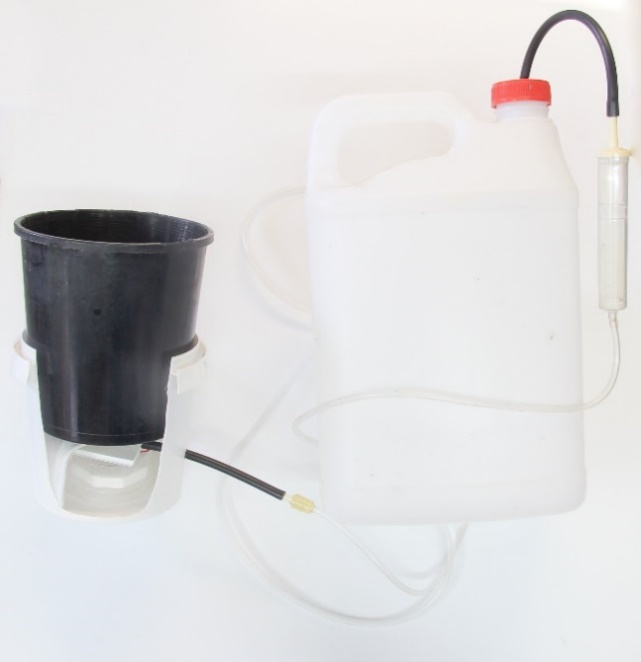

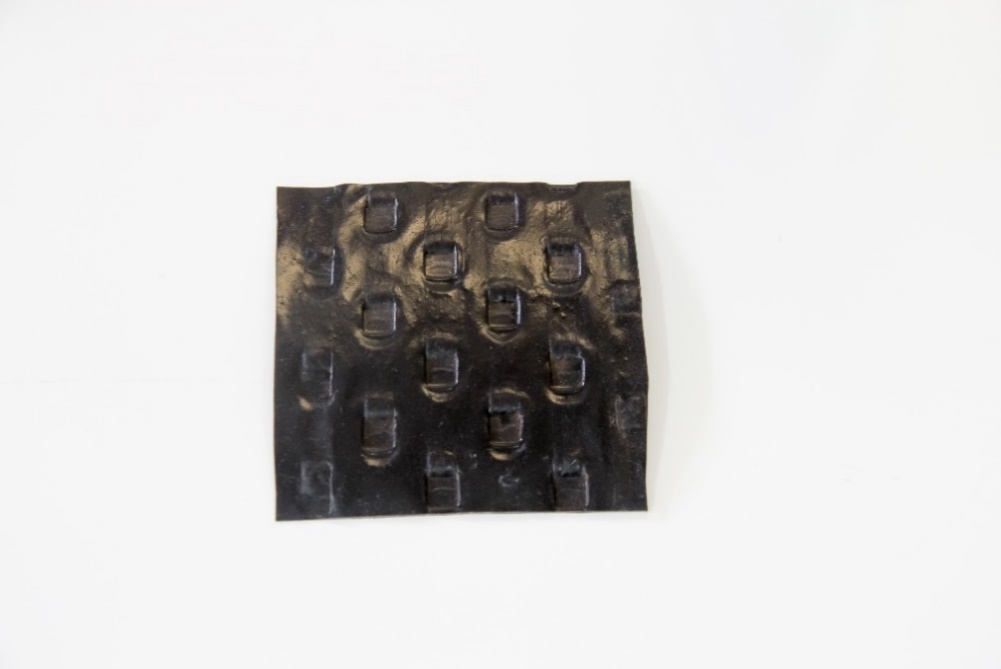


G

E

D

F

A

B

C

H

Fine (0.4mmID) shortened pipette tip inserted and glued into elbow joiner

Elbow joiner

Epoxy glue seals pipette tip in place

**Figure S2 Shoot transpiration efficiency over thermal time for all 11 genotypes studied.**

Figure S3 Genotypic variations in shoot transpiration efficiency measured at ~1000^o^Cd after sowing for all 11 genotypes studied.

A

B

*r^2^* = 0.51

*r^2^* = 0.002

*r^2^* = 0.42

C

D

*r^2^* = 0.83

*r^2^* = 0.90

E

F

*r^2^* = 0.83

*r^2^* = 0.89

H

G

*r^2^* = 0.056

**Figure S4 Comparisons of plant biomass and transpiration efficiency from (non-destructive) pot weight versus direct biomass measurements.**
